# A Cytological Study of Palpable Superficial Nodules of Parasitic Origin: A Study of 41 Cases

**DOI:** 10.1155/2014/373472

**Published:** 2014-03-17

**Authors:** Prashant Goyal, Shelly Sehgal, Soumyesh Ghosh, Deepti Mittal, Awanindra Kumar, Sompal Singh

**Affiliations:** Department of Pathology, Swami Dayanand Hospital, Dilshad Garden, Delhi 110095, India

## Abstract

*Background*. Few parasitic infestations present as only superficial palpable subcutaneous or intramuscular nodule. The current study highlights the role of FNAC in the diagnosis of superficial palpable parasitic lesions. *Methods*. This was a retrospective study in which we reviewed the FNAC record of all patients over a period of two years from September 2011 to August 2013. During this period, FNA was performed on 5954 cases which presented as superficial palpable lump at various sites of body. There were 41 cases diagnosed as parasitic lesion or suspicious of parasitic lesion on cytology which were included in the study. *Results*. In the present study, most of the patients were children and young adults. The lesions were located over trunk in 18 (43.9%) cases, extremities in 12 (29.3%) cases, and head and neck region in 11 (26.8%) cases. Out of 41 cases, 27 (65.8%) cases were confirmed on cytology and/or histopathology as parasitic lesions, including 21 (51.2%) cases of cysticercosis, 5 (12.2%) cases of filariasis, and one (2.4%) case of hydatid cyst. Cytological findings of remaining cases were suggestive of parasitic lesion. *Conclusion*. Careful assessment of cytological material is helpful to detect parasite or inflammatory response to parasite even in asymptomatic patients.

## 1. Introduction

Parasitic infestation is a serious health problem in developing countries. Some parasitic infestations do not present with any other symptoms except single or multiple superficial palpable nodules. Subcutaneous or intramuscular palpable parasitic nodules are most commonly due to cysticercosis [1]. Most of these cases are clinically misdiagnosed as benign or malignant soft-tissue tumor or lymphadenitis depending on the site [2]. So, most of the time, diagnosis of parasitic lesion on cytology is an incidental finding. Fine needle aspiration cytology (FNAC) is simple, sensitive, cost-effective, and rapid diagnostic tool for evaluation of any superficial palpable lump over various sites of the body [3]. The diagnostic role of FNAC in cysticercosis was first emphasized by Kung et al. in 1989 [4]. Since then FNAC has become a pivotal tool in evaluating subcutaneous and muscle nodules caused by parasites [2]. FNAC also obviates the need for a subsequent histopathological examination, as the parasites may not be demonstrated even on biopsy examination [3].

In our study, the relevant clinical data, gross findings, cytomorphological findings, histopathological findings, and treatment response, wherever available, were evaluated. This study illustrates the value of careful screening of aspirated cytological material which can render definitive diagnosis of clinically unsuspected cases of parasitic infestation.

## 2. Material and Methods

This was a retrospective study in which we reviewed the FNAC record of all patients over a period of two years from September 2011 to August 2013 at the Department of Pathology of our hospital, a multispecialty Government Hospital catering low to middle class population. 5954 patients, who had superficial palpable lump, underwent FNAC during this period. Out of these, 41 cases (0.68%) were diagnosed as parasitic lesion or suspicious of parasitic lesion. FNAC was performed by pathologist with a 22 or 23 gauge needle attached to a 20 mL disposable syringe by free hand conventional method of aspiration. The aspirated cytological material was smeared on the glass slides. In cases of fluid aspirate from cystic lesion, smears were prepared from cystic fluid after cytocentrifugation. The air-dried smears were stained with May-Grunewald-Giemsa (MGG) stain after fixation in methanol. All cytological slides were reviewed by two independent pathologists. The evidence of parasite or fragment of parasite was noted along with other cytomorphological features. Subsequent excision biopsy and response to medical treatment was also evaluated whenever possible and findings were correlated with the cytological findings. The age and sex of patient, size, site, clinical diagnosis of nodule, and nature of aspirate were also noted (Table 1).

## 3. Results

**Figure 1:**
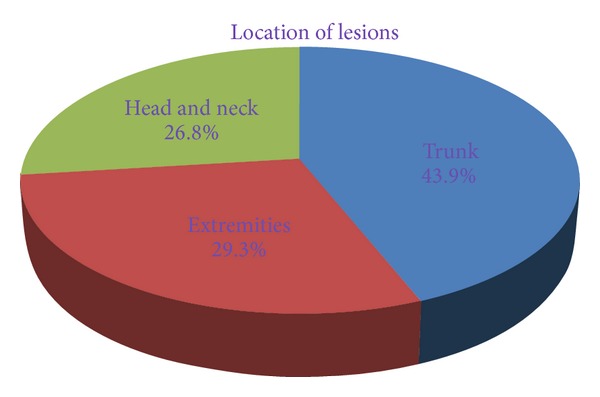
Distribution of lesions according to location.

**Figure 2:**
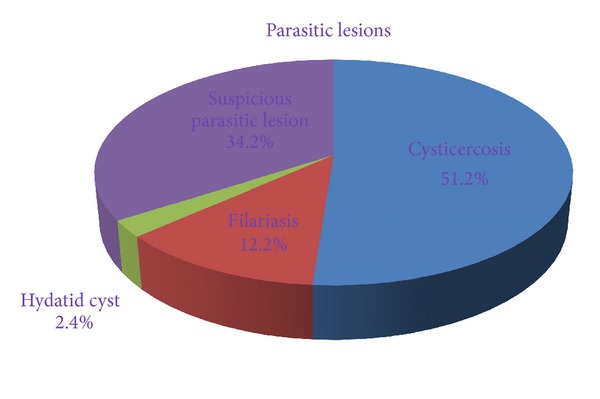
Distribution of parasitic lesions according to etiology.

We performed a total 5954 FNA of superficial palpable lumps from various sites during the study period and 41 cases (0.68%) were diagnosed as parasitic lesion or suspicious of parasitic lesion on cytology. Among 41 patients, 19 (46%) were males and 22 (56%) were females and presenting age ranged from 4 years to 38 years (mean age 18.9 years). All patients except two patients, presented with single, painless, superficial, slow-growing nodule of variable sizes while two patients presented with multiple nodules. On local examination, all the swellings were soft to firm, nontender, and nodular. In all the cases except one case of hydatid cyst, the diameter of swellings ranged from 0.5 to 4.5 cm (median 1.5 cm) while that of hydatid cyst was 11 cm. The lesions were located over trunk (including abdominal wall, chest, and back) in 18 (43.9%) cases, extremities in 12 (29.3%) cases, and head and neck in 11 (26.8%) cases (Figure 1). Provisional clinical diagnosis of parasitic lesion was made in only 2 (4.9%) cases, while the majority were clinically misdiagnosed as lipoma, neurofibroma, lymphadenitis, cold abscess, epidermal inclusion cyst, fibroadenoma, inflammatory lesion, and soft-tissue tumor. The aspirated material consisted of few drops to 5 mL fluid of variable consistency including clear fluid with or without granular deposit in 28 (68.3%) cases, purulent fluid in 10 (24.4%) cases, and blood-mixed fluid in 3 (7.3%) cases. No allergic reaction or any other complication was observed after the procedure. Out of 41 cases, 27 (65.8%) cases were confirmed on cytology and/or histopathology as parasitic lesions, included 21 (51.2%) cases of cysticercosis, 5 (12.2%) cases of filariasis, and one (2.4%) case of hydatid cyst (Figure 2).

### 3.1. Pathological Findings

**Figure 3:**
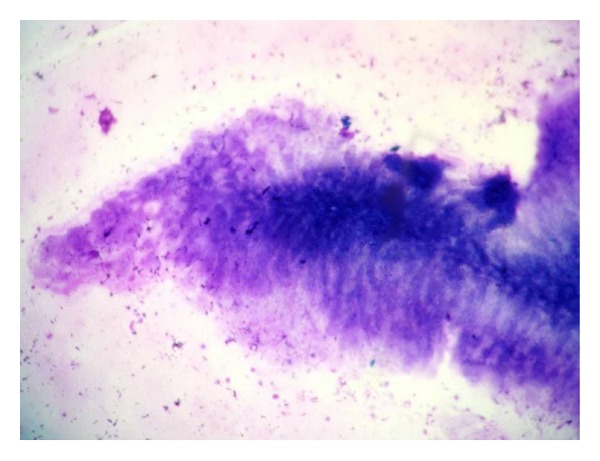
Cytological smear showing bladder wall of cysticercus cellulosae surrounded with mild inflammatory response (MGG stain, 60x).

**Figure 4:**
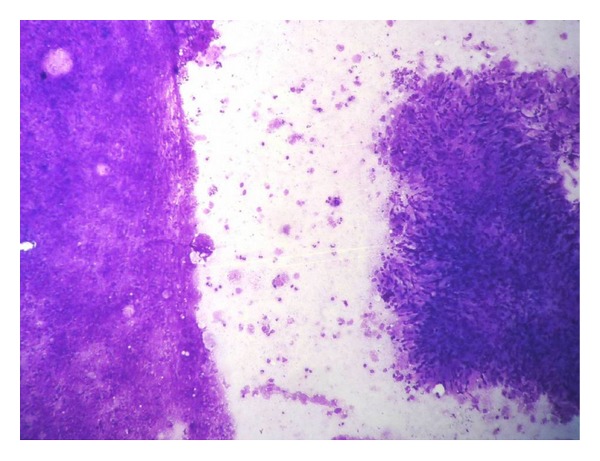
Cytological smear showing bladder wall of cysticercus cellulosae and epithelioid cell granuloma with palisading histiocytes (MGG stain, 40x).

**Figure 5:**
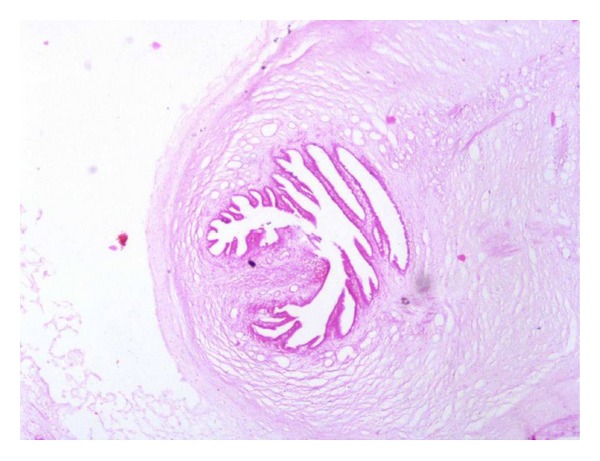
Histopathological section showing cysticercus cellulosae (H&E stain, 10x).

**Figure 6:**
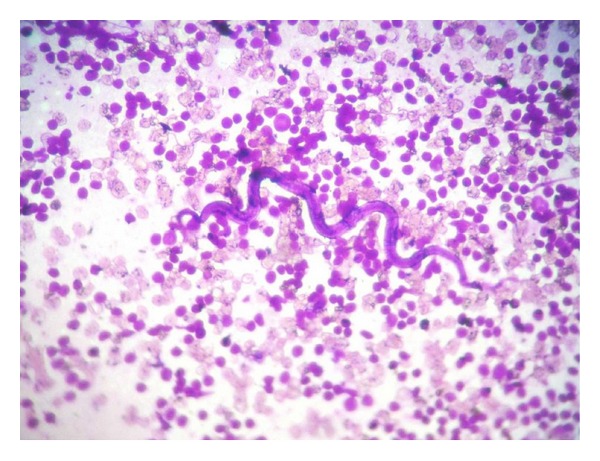
Cytological smear showing microfilaria surrounded with inflammatory cells and absence of eosinophil (MGG stain, 100x).

**Figure 7:**
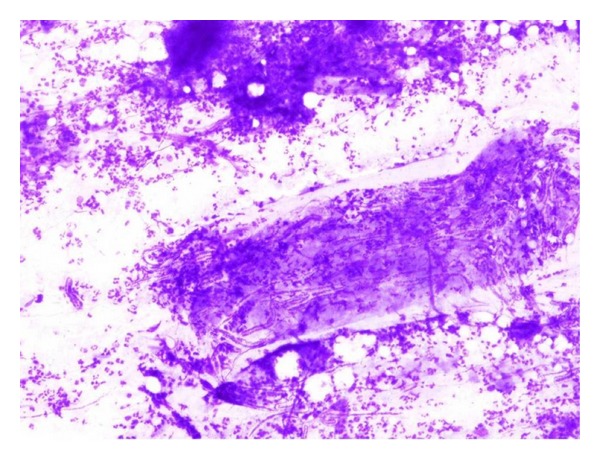
Cytological smear showing adult gravid filarial containing many microfilariae and surrounded with marked inflammatory cells (MGG Stain, 10x).

**Figure 8:**
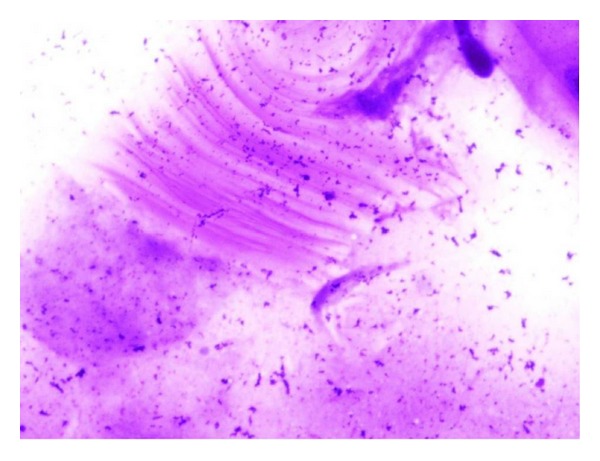
Cytological smear showing fragment of acellular lamellate membrane (MGG stain, 40x).

In 19 (46.3%) cases, a definite evidence of cysticercosis was observed in form of fragments of cysticercus bladder wall on cytology (Figure 3) while none of them showed hooklets or scolex. All these cases showed varying proportion of inflammatory response with or without giant cells and granuloma (Figure 4). Out of these 19 cases, histopathological correlation was available only in 2 cases which correlated with cytodiagnosis (Figure 5). The rest of the cases responded to medical treatment for cysticercosis. All 5 cases of filariasis showed microfilaria along with mild to severe mixed inflammatory response (Figure 6) while one case also showed gravid adult filarial worm in cytology (Figure 7). Thick and thin blood smear examination of nocturnal venous blood revealed no microfilariae in all 5 cases. One case which was diagnosed as hydatid cyst showed acellular lamellated membrane on cytology and was confirmed on histopathology (Figure 8).

No parasite or fragment of parasite could be seen on cytology in 16 (39%) cases, but in these cases, the cytological findings were highly suggestive of a parasitic lesion. The cytology of these cases showed mild to moderate mixed inflammatory infiltrate, histiocytes, multinucleated giant cells, and epithelioid noncaseous granuloma in a dirty granular background. None of these cases showed hooklets or scolex on cytology. In 3 of these cases, a histopathological correlation was available, which revealed definite parasitic element of cysticercosis in 2 cases and the remaining case was reported as suggestive of parasitic lesion in view of absence of any parasitic element.

In this study, all cases of parasitic lesions or suspicious of parasitic lesion were associated with varying degree of mixed inflammatory response ranging from few histiocytes to marked mixed inflammatory cell infiltrate. Eosinophil was seen in only 13 (32%) out of the 41 cases while lymphocytes, histiocytes including palisading histiocytes, neutrophil, foreign body giant cells, and epithelioid granuloma were seen in 32 (78%), 28 (68%), 20 (49%), 16 (39%), and 9 (22%) cases, respectively. Dirty granular background was seen in 12 (29%) cases (Table 1).

## 4. Discussion

The palpable superficial parasitic nodules are often clinically misinterpreted as benign or malignant mesenchymal tumors or as lymphadenopathy. FNAC has emerged as a widely acceptable method for the diagnosis of parasitic lesion. In the present study, most of the patients were children and young adults with almost equal sex distribution. Cysticercosis was found to be the most common cause of superficial palpable parasitic nodules in previous studies as in our study [1, 5]. Other parasites identified were filaria and* Echinococcus*.

Cysticercosis in humans is an ancient disease and has even been detected in Egyptian mummies by paleoparasitologists [6]. Cysticercosis is the larval stage infection of the cestode* Taenia solium*. Humans are the only definitive host and can also act as intermediate hosts by ingestion of raw or poorly cooked vegetables or water contaminated with eggs or pork infested with larvae [7]. Cysticercosis is also common in vegetarians due to lack of basic sanitation facilities in developing countries [8]. Though cysticercus can be found in any organ, it commonly manifests as subcutaneous and intramuscular nodules which often are clinically misdiagnosed as inflammatory or mesenchymal lesions like in our study [8]. Essential for the cytodiagnosis of cysticercosis is identification of the parasitic fragments including its bladder wall and hooklets. Parasitic fragments may comprise bluish, fibrillary structures, sometimes with honeycombing, calcospherules, tegument thrown into rounded wavy folds, scolex with hooklets, and hyaline membrane surrounding it [9–11]. The physical factors such as the firm nonexpansile nature of the host tissue may limit the growth of the parasite and initiate the host inflammatory response. The presence of eosinophils, neutrophils, palisading histiocytes, giant cells, and atypical granular dirty background in an aspirate from a subcutaneous nodule should alert the cytopathologist of a parasitic infestation. Nonetheless, still in some cases of cysticercosis, none of these features may be present, and the inflammatory infiltrate may also be variable [2]. Finding an entire scolex in FNA is a rare event [12]. None of our cases showed hooklets or scolex on cytology.

Filariasis is a major public health problem in many tropical and subtropical countries and is prevalent in India, China, Indonesia, Africa, and the Far East. It is transmitted by the Culex mosquito and is caused by two closely related nematodes:* Wuchereria bancrofti* and* Brugia malayi*.* W. bancrofti* is responsible for 90% cases of filariasis [13]. All 5 cases of filariasis in our study were also caused by* W. bancrofti*. The majority of patients with filariasis are asymptomatic and diagnosed incidentally. Microfilaremia and eosinophilia are common in the acute phase. The various sites where microfilaria can be detected are thyroid, breast, skin and soft tissue swellings, epididymis, salivary glands, lymph nodes, urine, endoscopic brushings, and effusion fluids [14, 15]. In the present study, none of the patients was clinically suspected of filariasis; clinically they presented with breast lump (2 cases) and lymphadenopathy (3 cases). All cases showed mild to severe inflammatory reaction. In our study, only two cases had eosinophil in the aspirate; hence, eosinophils are not necessarily seen in lesions of filariasis.

Hydatid disease, also known as hydatosis or echinococcosis, is most frequently caused by* E. granulosus* and the commonly affected organs are liver and lung. This disease is endemic in cattle and sheep rearing regions of the world. The majority of cases of HCs are asymptomatic although clinical signs and symptoms depend on the anatomic location, size, and pressure effect of growing cysts. Therefore, the signs and symptoms are variable and never pathognomic of HC [16]. So the majority of HC in subcutaneous or rare locations are misdiagnosed on clinical examination. Superficial HC usually presents as slow growing, fluctuant, and painless mass [16]. HC is usually not included in the differential diagnosis of subcutaneous or superficial palpable mass due to its rarity, even in endemic areas. The presence of a laminated membrane with parallel striations, dispersed retractile hooklets, granular debris, and multinucleated giant cells is consistent with the diagnosis of a HC [17]. FNAC is not recommended in suspected case of HC because of possibility of acute anaphylactic reaction due to spillage of hydatidfluid. However, some authors have reported that no complications were encountered during FNAC procedure as in our study [18].

In our study, the nature of aspirate was clear fluid with or without granular deposits in majority of cases. Hence, such clear fluid aspirate should alert the cytopathologist towards the possibility of parasitic etiology. In the majority of cytosmears, we found varying degree of inflammatory response comprising of histiocytes including palisading histiocytes and mixed inflammation including eosinophil, giant cells, and granulomas. Similar cytological features have been reported by other series also, although presence of eosinophils was relatively more common associated cytological feature as compared to our study [19].

## 5. Conclusion

Parasitic etiology is an important differential diagnosis of soft tissue nodule and FNAC is rapid, safe, cheap, and reliable diagnostic tool for such lesions. Careful assessment of cytological material is helpful to detect parasite or inflammatory response to parasite even in asymptomatic patients. The spectrum of host response may vary from no reaction to a marked inflammatory response. The entire spectrum of changes should be kept in mind while practicing cytopathology in an endemic area. In such situations, a high index of suspicion and careful screening of cytology smears are keys to a correct diagnosis.

## Figures and Tables

**Table 1:** Clinical data and cytological features of all the cases included in present study.

Cytological diagnosis	No. of cases	Age range (mean) year	Sex	Size range (median) cm	Distribution of different cytological findings							
					N (%)	L (%)	E (%)	H (%)	PH (%)	GC (%)	G (%)	DGB (%)
Cysticercosis	19	4–34 (16.8)	M: 9 F: 10	0.5–3.0 (1.5)	10 (53)	16 (84)	7 (37)	14 (73)	6 (31)	9 (47)	6 (31)	6 (31)
Filariasis	5	10–32 (20.8)	M: 2 F: 3	1.0–2.5 (1.0)	2 (40)	4 (80)	2 (40)	3 (60)	0 (0)	1 (20)	1 (20)	0 (0)
Hydatid cyst	1	35	M: 1 F: 0	11	0	0	0	0	0	0	0	1 (100)
Suspicious parasitic lesion	16	5–38 (19.7)	M: 7 F: 9	0.5–4.5 (1.5)	8 (50)	12 (75)	4 (25)	11 (69)	5 (31)	6 (37)	2 (13)	5 (31)

Total	41	4–38 (18.9)	M: 19 F: 22	0.5–11 (1.5)	20 (49)	32 (78)	13 (32)	28 (68)	11 (27)	16 (39)	9 (22)	12 (29)

M: male; F: female; N: neutrophil; L: lymphocytes; E: eosinophil; H: histiocytes; PH: palisading histiocytes; GC: giant cells; G: granuloma; DGB: dirty granular background.
